# Remodelling of Humeral Fracture in a Child with Osteopetrosis Tarda following Conservative Management - A 7-year Follow-up: A Case Report

**DOI:** 10.5704/MOJ.1907.008

**Published:** 2019-07

**Authors:** S Erkus, A Turgut, O Kose, O Kalenderer

**Affiliations:** Department of Orthopaedics and Traumatology, Izmir Tepecik Training and Research Hospital, Izmir, Turkey; * Department of Orthopaedics and Traumatology, Antalya Training and Research Hospital, Antalya, Turkey

**Keywords:** osteopetrosis tarda, fractures, remodeling, conservative treatment, children

## Abstract

Osteopetrosis (OP) is a rare hereditary sclerosing bone dysplasia characterised by generalised hard and brittle bone secondary to defective osteoclastic function. Osteopetrotic bone is brittle, thus these subjects are prone to frequent fractures, particularly of the long bones. Due to defective osteoclastic function, remodeling is also defective in OP. This report is a case of humeral fracture in a 9 years old girl who was followed seven years. The fracture had remodeled totally similar to healthy bone at the final follow-up. Conservative treatment should be kept in mind in the management of fractures in children with OP, and fractures within acceptable angulations and/or translations should be treated conservatively without hesitation.

## Introduction

Osteopetrosis (OP) is a rare hereditary sclerosing bone dysplasia characterised by generalised hard and brittle bone secondary to defective osteoclastic function. Three distinct subtypes exist; (i) infantile malignant, (ii) intermediate and (iii) benign (late-onset)^1^. The infantile (malignant) form has an autosomal recessive inheritance pattern and follows a fatal clinical course and patients usually die within the first decade of life due to bone marrow suppression, pancytopenia, hepatosplenomegaly, immunodeficiency and life-threatening infections. The intermediate autosomal recessive form carries life expectancy into adulthood. This type has the highest incidence of osteomyelitis of the jaw attributed to the decreased bone vascularity and the relatively low white blood cells^1^. The benign autosomal dominant osteopetrosis (ADO), also known as osteopetrosis tarda, is the most common. Most patients with ADO have normal life span, and seem otherwise healthy. An estimated 40% of patients with ADO are asymptomatic^2^.

Because the osteopetrotic bone being brittle in patients with ADO, they are prone to frequent fractures, particularly of the long bones. The treatment of fractures in osteopetrosis can be complicated and difficult. Surgical treatment of osteopetrotic fractures is associated with significant difficulties and pitfalls, including extreme resistance to drilling and cutting as a result of the hardness of the bone, hardware failure, peri-prosthetic fractures, refractures, delayed union, pseudarthrosis and infection. Although there are several case reports and clinical studies on the surgical treatment of ADO, there are only few cases regarding non-surgical treatment of paediatric fractures in ADO^3-5^.

We report a 7-year follow-up of a paediatric patient with ADO who sustained a humeral fracture and was treated non-surgically. Although, it has been proposed that the remodeling process is defective in ADO, we observed almost normal remodeling in long-term follow-up in our patient.

## Case Report

**Fig. 1:**
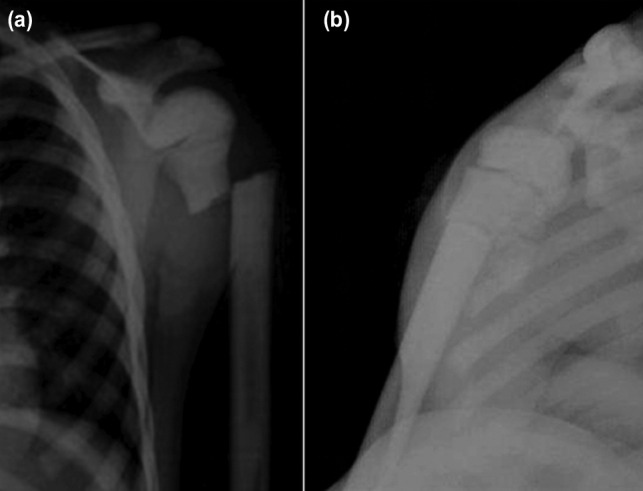
(a) Anteroposterior and (b) transthoracic lateral shoulder radiographs of the patient at initial admission. Note the pattern of fracture (transverse) and the diffuse sclerosis of humeral bone without an intramedullary canal.

A nine years old girl was seen in our emergency department with left shoulder pain, swelling and visible deformity after a simple ground level fall on her shoulder. She was right hand dominant. On physical examination, there was crepitation and abnormal movement over the shoulder. The passive movements of the shoulder were painful. Neurovascular examination was normal. She was otherwise a healthy child without a known systemic disease or abnormality. Radiographs of left shoulder demonstrated a transverse fracture at the proximal humeral diaphysis (Fig. 1).

After obtaining informed consent from the patient and parents, and approval of the institutional and national research committee, treatment was started.

**Fig. 2:**
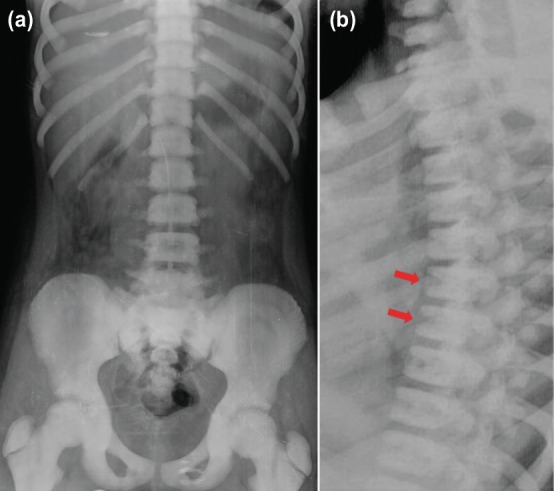
(a) Anteroposterior and (b) lateral spinal column radiographs. The red arrows indicate typical sandwich vertebrae.

Under sedation, closed reduction of the fracture was performed and the arm was immobilised with a Velpaue bandage. Post-reduction radiographs showed acceptable quality of reduction. Because the radiographic appearance of the bones was sclerotic, the pattern of the fracture was transverse and the intramedullary canal not visualised, hereditary metabolic bone diseases, particularly osteopetrosis tarda was suspected. The patient was admitted and further radiographic, laboratory and genetic investigations were performed. Plain radiographs of the spinal column showed 'sandwich vertebrae' appearance, which was a typical radiographic finding in osteopetrosis (Fig. 2). Routine laboratory tests were normal except for anaemia (Hgb 11.2gr/dl, normal range 12-16gr/dl). Genetic testing provided the definitive diagnosis of benign late onset osteopetrosis with mutation in chloride channel 7 (CLCN7) gene.

**Fig. 3:**
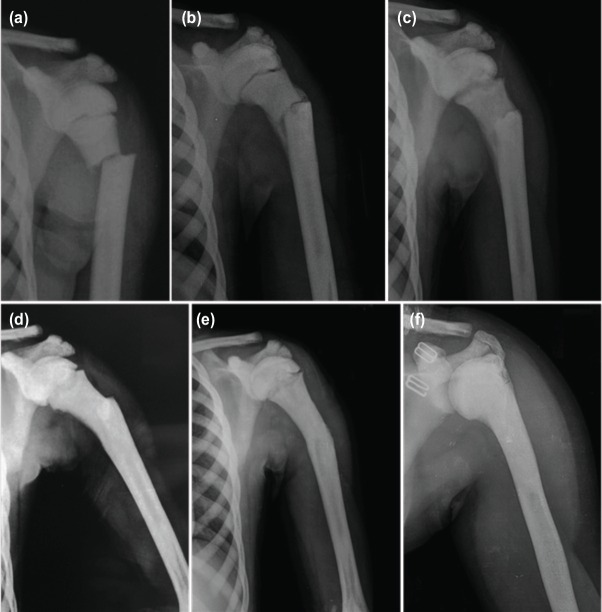
Serial radiographs of the patient’s through-out the follow-up controls at (a) initial admission, (b) 1 month, (c) 2 months, (d) 6 months, (e) 48 months and (f) at the final follow-up seven years after the initial injury. The remodeling of fracture is clearly seen.

The patient was discharged and followed-up with regular shoulder radiographs. Immobilisation with Velpaeu bandage was discontinued four weeks later after which shoulder range of motion exercises were started. At the end of eight weeks the fracture had united and the patient was free of pain with normal shoulder range of motion (ROM) and function. Thereafter, the patient was followed-up yearly. At the final follow-up seven years after the injury, the fracture had remodeled to nearly normal shape and alignment (Fig. 3).

## Discussion

Frequent fractures are a typical presentation of osteopetrosis tarda, and may be the first presentation in a previously asymptomatic patient similar to the present case. The classic radiological characteristics of ADO include diffuse sclerosis, affecting the skull, spine, pelvis and appendicular bones, focal sclerosis of the skull base, pelvis and vertebral end plates – "sandwich" vertebrae^1,4^. The fracture pattern in tubular bones is usually transverse. In case of such radiographic findings in a paediatric patient ADO should be suspected.

Because there is a genetic defect in the chloride channel 7 gene (CLCN7) which is a proton pump, osteoclasts lose their ability to acidify the Howship’s lacuna and to resorb bone and calcified cartilage. It is well-known that osteoclasts have a significant role in both fracture healing and remodeling. Thus, it is proposed that remodeling is also defective in ADO^1^. However, in the present case remodeling has been observed at the seventh year follow-up. It may be slower than healthy subjects, probably depending on the extent of deficiency in expression of the gene.

Due to difficulties and several complications with the surgical treatment of fractures in patients with ADO, conservative treatment should be chosen with proper indications, particularly in children. Armstrong *et al* reported successful union of distal radius fractures with conservative treatment in 11 patients^5^. Dahl *et al* treated multiple fractures in four patients with malignant osteopetrosis using casting and traction^4^. Finally, Krieg *et al* treated even femoral neck fractures with non-surgical treatment in two patients with ADO^3^. All these previous reports demonstrate that conservative treatment can be successful for management of fractures in osteopetrosis, and fracture union is usually achieved.

In conclusion, although previously proposed that remodeling is defective in ADO, our case showed that remodeling can be achieved similar to healthy bone with time. Therefore, non-surgical treatment should be kept in mind in the management of fractures in children with ADO. During decision making, fractures within acceptable angulations and/or translations should be treated without surgery and without hesitation.
